# MRA_1571 is required for isoleucine biosynthesis and improves *Mycobacterium tuberculosis* H37Ra survival under stress

**DOI:** 10.1038/srep27997

**Published:** 2016-06-29

**Authors:** Rishabh Sharma, Deepa Keshari, Kumar Sachin Singh, Shailendra Yadav, Sudheer Kumar Singh

**Affiliations:** 1 Microbiology Division, CSIR-Central Drug Research Institute, B.S. 10/1, Sector 10, Jankipuram Extension, Sitapur Road, Lucknow-226031, India; 2 Academy of Scientific and Industrial Research (AcSIR), New Delhi, India

## Abstract

Threonine dehydratase is a pyridoxal 5-phosphate dependent enzyme required for isoleucine biosynthesis. Threonine dehydratase (IlvA) participates in conversion of threonine to 2-oxobutanoate and ammonia is released as a by-product. MRA_1571 is annotated to be coding for IlvA in *Mycobacterium tuberculosis* H37Ra (*Mtb*-Ra). We developed a recombinant (KD) *Mtb*-Ra strain by down-regulating IlvA. The growth studies on different carbon sources suggested reduced growth of KD compared to wild-type (WT), also, isoleucine concentration dependent KD growth restoration was observed. The expression profiling of IlvA suggested increased expression of IlvA during oxygen, acid and oxidative stress. In addition, KD showed reduced survival under pH, starvation, nitric oxide and peroxide stresses. KD was more susceptible to antimycobacterial agents such as streptomycin (STR), rifampicin (RIF) and levofloxacin (LVF), while, no such effect was noticeable when exposed to isoniazid. Also, an increase in expression of IlvA was observed when exposed to STR, RIF and LVF. The dye accumulation studies suggested increased permeability of KD to ethidium bromide and Nile Red as compared to WT. TLC and Mass studies confirmed altered lipid profile of KD. In summary down-regulation of IlvA affects *Mtb* growth, increases its susceptibility to stress and leads to altered cell wall lipid profile.

Tuberculosis caused by *Mycobacterium tuberculosis* (*Mtb*) remains a major health concern as approximately 9 million new cases of tuberculosis (TB) were reported during year 2013^1^. In addition, 1.5 million people died due to TB during that period. With the increasing incidences of MDR and XDR-TB infections being documented in clinical settings, this remains a high priority area of research for new chemotherapies. Metabolic pathways essential for growth and survival of *Mtb* make attractive targets for growth inhibition. *Mtb* has evolved to survive inside the host macrophages^2^,^3^. Host macrophages contain *Mtb* in a nutritionally poor environment and *Mtb* needs to survive by synthesizing most of the metabolites required for its growth^4^. Biosynthesis of amino acids may become an important growth regulator in the absence of its availability from the host. The essentiality of several amino acid biosynthetic pathways had already been demonstrated by developing recombinants of amino acid biosynthetic pathways^5^,^6^,^7^,^8^,^9^,^10^.

Branched-chain amino acids (BCAAs) - isoleucine, valine and leucine biosynthetic process is lacking in humans and a number of BCAAs auxotrophs from *Mycobacteria tuberculosis*, *Streptococcus pneumoniae, Actinobacillus pleuropneumoniae, Burkholderia pseudomallei, and Haemophilus influenzae* have been studied as immunizing agents^8^,^11^,^12^,^13^,^14^. Also, knockout strains of acetohydroxyacid synthase (*ilvB1*) were attenuated for growth and virulence in mice^15^. L-threonine acts as a precursor for L-isoleucine and flux from threonine to isoleucine is under allosteric control of threonine dehydratase and acetohydroxy acid synthase (AHAS). In addition, there is regulation at transcription level for genes encoding AHAS enzymes^16^. In bacteria and fungi, homoserine, the last common precursor for threonine and methionine is feedback inhibited by threonine^17^,^18^. Threonine can be metabolized through glycine and in this process; it produces an acetyl-CoA molecule that enters TCA cycle. The glycine can further move through glycine cleavage system generating precursors for one carbon metabolism and reducing equivalents or be converted into serine and in the process can generate THF^19^. Threonine dehydratase (IlvA) is the first enzyme for biosynthesis of isoleucine. The threonine dehydratases function as non-oxidative deaminators of threonine and/or serine and release alpha-ketobutyrate and pyruvate as products, respectively, along with release of ammonia. This ammonia may be used in transamination reactions or as a buffering agent in slightly acidic environment of phagosome. It reacts with H^+^ ions and changes into ammonium ions (NH_4_^+^) that resists acid stress during mycobacterial infections^20^. Threonine dehydratase mediated isoleucine biosynthesis is an important step in maintaining the metabolic pool of isoleucine, a branch chain amino acid. Branch chain amino acids play an important role in proteins structural conformations with isoleucine and valine being present in β-sheets while leucine playing a role in α-helices and leucine zippers^21^. Given the possibilities for threonine metabolism and its possible role in isoleucine synthesis as well as its essentiality for *in vitro* growth^22^, we studied the role of MRA_1571, annotated to be a threonine dehydratase (IlvA) during *Mtb* growth and survival.

## Results

### Development of knockdown strain

The knockdown strain was developed using antisense technique. This relies on the binding of antisense strand to the sense transcript being transcribed from sense strand. This binding interferes with subsequent RNA translation and leads to an overall decrease in target protein levels in the cell. The knockdown strain was confirmed by PCR amplification of genomic DNA using primers from IlvA gene reverse (primer-1) and *aph* reverse (primer-2). PCR amplification of ~2.3 kb, similar to calculated size (Supplementary Fig. S1) was observed. The amplicon’s digestion with *Not*I restriction enzyme led to the expected release of approximately ~674 bp fragment. The expression down-regulation was confirmed by immunoblotting and transcript analysis with close to 60% down-regulation being observed by protein abundance analysis (Supplementary Fig. S2).

### Effect of carbon source on growth

**Figure 1:**
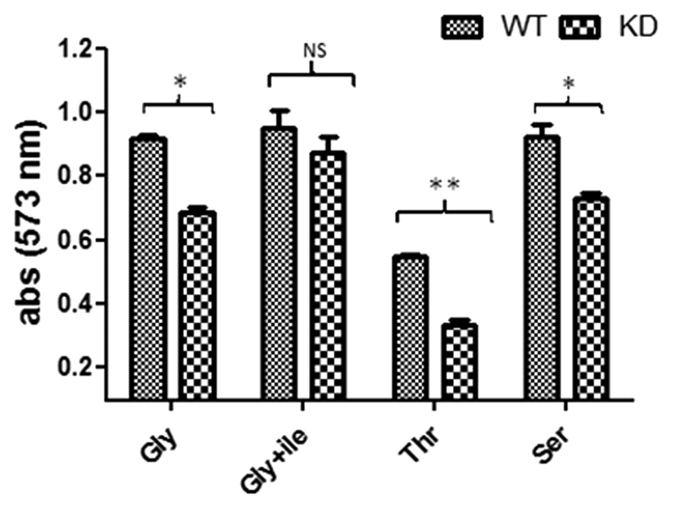
Effect of carbon source on growth. MABA based growth studies of wild-type (WT) *Mycobacterium tuberculosis* H37Ra and IlvA knockdown (KD) were performed on different carbon sources. Sauton’s medium without glycerol was used as a basal medium and glycerol (Gly), glycerol+isoleucine (1 mM) (Gly+ile), threonine (Thr), and serine (Ser) were used as a carbon source. Glycerol was used @ 3% while threonine and serine were used @ 1%. Results represent the mean ± SEM from three independent experiments performed in triplicate and significance analysis was done by Student’s *t*-test, ^NS^*p* > 0.05, *p < 0.05, **<0.01.

The growth of wild-type (WT) and recombinant strain (KD) was measured by MABA assay using different carbon sources. Growth of KD was less in comparison to WT on glycerol as a carbon source; however, KD growth restoration occurred after isoleucine supplementation. Although, growth on threonine was less for both WT and KD, however, growth difference between two was substantial with KD showing lesser growth than WT. KD was also slow to use serine as a carbon source compared to WT (Fig. 1). These data suggest that down-regulation of IlvA makes *Mtb*-Ra defective in growth and growth can be complemented by addition of isoleucine. To further study the effect of isoleucine concentration on KD growth, another experiment with isoleucine supplementation varying from 0.25 mM to 4 mM was performed and isoleucine concentration dependent growth restoration of KD was observed (Supplementary Fig. S3). Growth of WT was not affected by isoleucine supplementation.

### Effect of oxygen stress on expression

**Figure 2:**
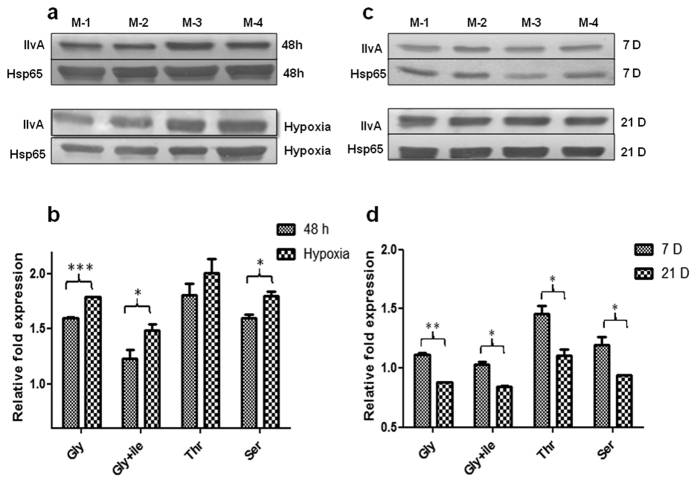
Immunoblotting studies. Expression of IlvA was studied in WT *Mycobacterium tuberculosis* H37Ra on different carbon sources. (**a**) Immunoblot of IlvA from WT at 48 h and under hypoxia. (**b**) Densitometry analysis showing relative fold expression of IlvA from WT at 48 h and under hypoxia. (**c**) Immunoblot of IlvA from WT after 7 days and 21 days of growth. M-1, M-2, M-3 and M-4 refer to glycerol (Gly), glycerol+ isoleucine (1 mM) (Gly+ile), threonine (Thr), and serine (Ser) respectively. Glycerol was used @ 3%, while, threonine and serine were used @ 1%. (**d**) Densitometry analysis showing relative fold expression of IlvA from WT after 7 days and 21 days of growth. All densitometry data represent normalized ratio with Hsp65 used as a loading control. Results represent the mean ± SEM from three independent experiments performed in triplicate and significance analysis was done by Student’s *t*-test, *p < 0.05, **<0.01, ***p < 0.001.

For oxygen stress studies methylene blue (1.5 μg/ml) was used as a hypoxia indicator and a complete colour loss was taken as hypoxic condition^23^. The medium colour loss was gradual and affected by the carbon source used. This suggests differences in growth rate on different carbon sources. Complete loss of colour of WT growth medium varied and complete discolouration of medium occurred on 28^th^ day while using threonine as a carbon source. Also, WT growth on glycerol, glycerol + isoleucine and serine as carbon source showed complete discolouration by 21^st^ day. Similarly, hypoxia development in KD varied with media used and it was slow to develop in both serine (24 days) and threonine (30 days) as a carbon source. Hypoxia on glycerol as a carbon source and glycerol+ isoleucine showed loss of colour on 22^nd^ day in KD. The expression studies with WT showed considerably increased IlvA expression under hypoxic conditions as compared to expression after 48 h of growth on glycerol, glycerol + isoleucine and serine as a carbon source (Fig. 2a,b).

To further confirm the oxygen saturation status and its effect on IlvA expression, we used DosR expression as a marker for hypoxia. DosR is upregulated under hypoxic conditions^24^ and we performed expression profiling of IlvA and DosR by RT-PCR at 48 h and after complete loss of methylene blue colour. An up-regulation of both DosR and IlvA was observed (Supplementary Fig. S4), suggesting that loss of medium colour was also an indication of cells turning hypoxic and IlvA is up-regulated under hypoxic conditions.

The time dependent expression studies carried out at two time points of 7 days and 21 days under aerobic growth conditions showed a decrease in IlvA expression on 21^st^ day as compared to expression on 7^th^ day. Expression intensities were stronger on threonine as a carbon source on both time points (Fig. 2c,d).

### Effect of pH and starvation stress on expression and survival

**Figure 3:**
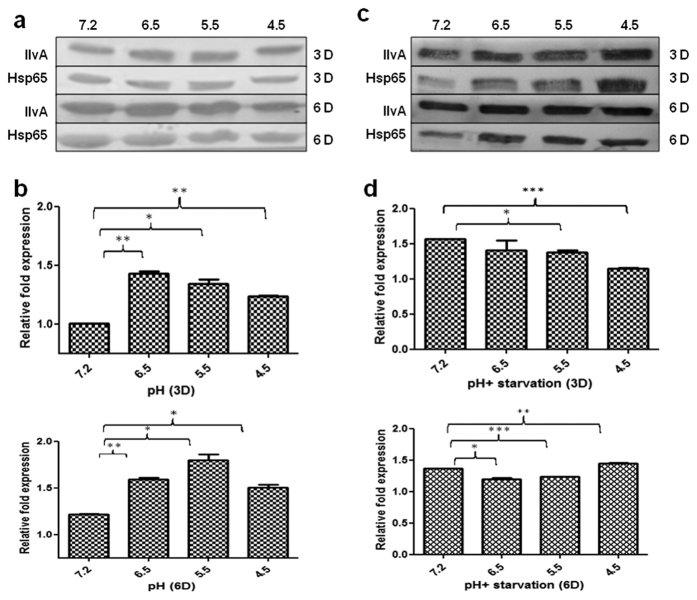
Immunoblotting studies under pH and starvation stress. IlvA expression studies in WT were performed under different pH and pH+ starvation stress conditions. (**a**) Immunoblot of IlvA at different pH values after 3 days and 6 days of growth. (**b**) Densitometry analysis showing relative fold expression of IlvA at different pH values after 3 days and 6 days. (**c**) Immunoblot of IlvA under different pH+ starvation stress after 3 days and 6 days. Experiments at different pH were performed using Sauton’s medium with glycerol (3%) as a carbon source. (**d**) Densitometry analysis showing relative fold expression of IlvA under different pH+ starvation stress after 3 days and 6 days. Experiments with combined pH and starvation stress were performed in phosphate citrate buffer respectively. All densitometry data represent normalized ratio with Hsp65 used as a loading control. Results represent the mean ± SEM from three independent experiments performed in triplicate and significance analysis was done by Student’s *t*-test, *p < 0.05, **<0.01, ***p < 0.001.

The expression studies of IlvA were performed from WT culture at different pH values. It showed an increase in expression after 72 h of growth at pH = 6.5 and pH = 5.5 compared to pH = 7.2 (Fig. 3a). In addition, expression levels remained substantially higher at pH = 6.5, 5.5 and 4.5 after 144 h, compared to expression of WT at pH = 7.2 (Fig. 3a,b). The expression intensities were also studied under combined (low pH + starvation) stress. Initially after 72 h, expression of IlvA was comparable at pH = 7.2 and 6.5, but low at pH = 5.5 and 4.5 (Fig. 3c,d). After 144 h, an increase in expression at pH = 4.5 was observed which was considerably more than the expression at pH = 7.2. However, expression levels at pH = 5.5 and 6.5 remained low when compared with pH = 7.2 (Fig. 3c,d).

To confirm whether the increased expression under low pH stress had something to do with the survival of *Mtb* under low pH and increased expression was a way of averting pH stress for improved *Mtb* survival. In addition, whether discordant expression at pH = 4.5 after 3 days and less increase in protein abundance compared to expression at pH = 6.5 and 5.5 was due to physiological changes taking place, we studied the survival of WT and KD at low pH as well as under dual stress of low pH and starvation.

**Figure 4:**
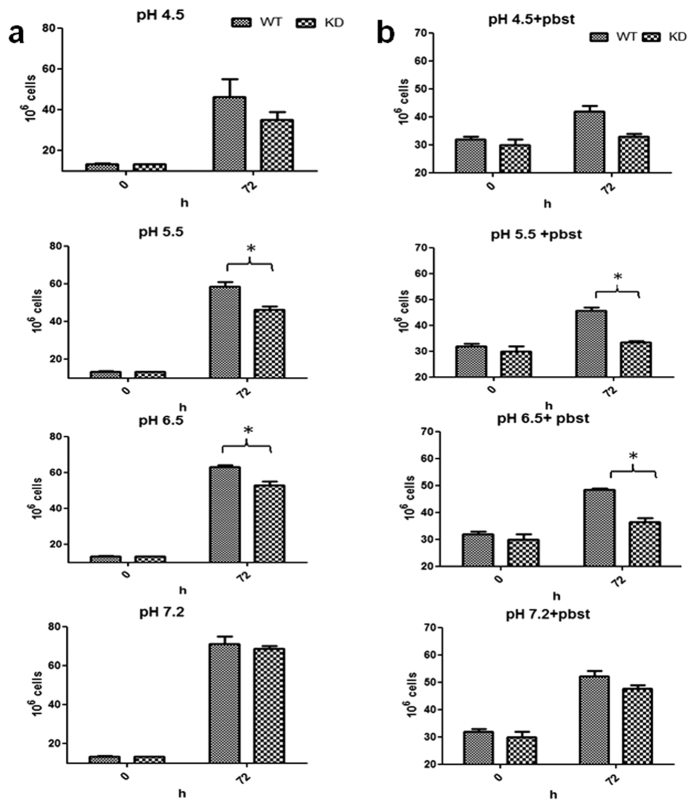
Survival studies under pH and starvation stress. Both WT and KD were subjected to (**a**) low pH (pH = 6.5, 5.5 and 4.5) and (**b**) low pH (pH = 6.5, 5.5 and 4.5) + starvation stress. In starvation conditions, no carbon or nitrogen source was provided and phosphate citrate buffer (pbst) was used for incubation at different pH values, while in pH stress alone, Sauton’s medium with glycerol as a carbon source was adjusted to different pH values. Results represent the mean ± SEM from three independent experiments performed in triplicate and significance analysis was done by Student’s *t*-test, *p < 0.05.

The survival of both WT and KD on pH = 7.2 was comparable after 72 h stress, however, considerable difference in survival of WT and KD was observed at acidic pH (pH = 5.5 and 6.5), with knockdown showing lower survival compared to WT (Fig. 4a). Similar results were observed under combined pH and nutritional starvation stress (Fig. 4b). However, survival of both, WT and KD on pH = 4.5, was comparable under both stress conditions.

### Effect of peroxide and nitric oxide stress on survival

**Figure 5:**
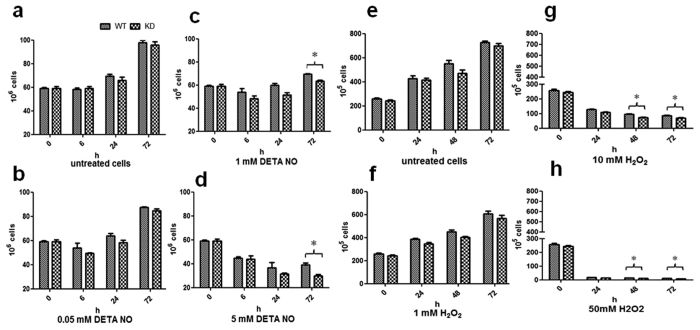
Effect of Nitric oxide and peroxide on survival. Both WT and KD were subjected to different concentrations of Hydrogen peroxide and DETA-NO and survival was estimated by CFU count. (**a–d**) growth of WT and KD performed after subjecting them to increasing concentrations of DETA-NO. (**a**) is untreated, (**b**–**d**) are treated with 0.05, 1 and 5 mM DETA-NO respectively. (**e–h**) growth of WT and KD performed after subjecting them to increasing concentrations of hydrogen peroxide, (**e)** is untreated, (**f**–**h**) are treated with 1, 10 and 50 mM hydrogen peroxide respectively. Results are mean ± SEM from three independent experiments performed in triplicate and significance analysis was done by Student’s *t*-test, *p < 0.05.

The survival studies under nitric oxide stress showed no effect at lower concentration of DETA-NO, but, substantial difference in survival of KD compared to WT, was observed at higher concentrations of DETA-NO. KD showed lower survival after 72 h compared to WT. This increased susceptibility to stress suggested KD being more susceptible to killing by DETA-NO than the WT (Fig. 5a–d). The survival study under peroxide stress also suggested a trend similar to the effect observed with nitric oxide. An increased killing of KD occurred at increasing hydrogen peroxide concentration compared to WT (Fig. 5e–h).

### Permeability studies

**Figure 6:**
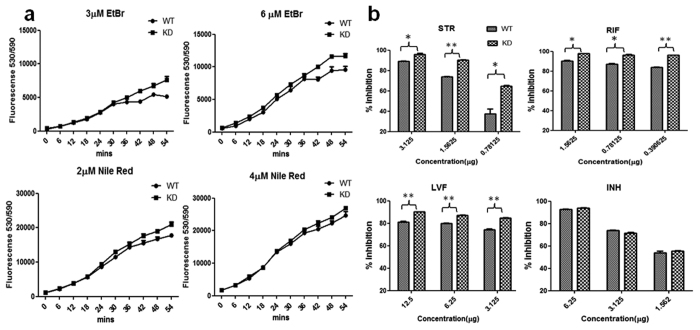
Effect of knockdown on cell permeability and susceptibility. (**a**) Whole cell uptake studies with EtBr and Nile Red were performed using both WT and KD. EtBr was studied at 3 and 6 µM concentrations, while, Nile Red was studied at 2 and 4 µM concentrations. (**b**) Susceptibility to antimycobacterial agents was studied with both WT and KD. Antimycobacterial agents studied were Streptomycin (STR), Levofloxacin (LVF), Rifampicin (RIF) and Isoniazid (INH). Results are mean ± SEM from three independent experiments performed in triplicate, significance analysis was done by Student’s *t*-test, *p < 0.05, **<0.01, ***p < 0.001.

To study whether enhanced susceptibility to peroxide and nitric oxide stress was a manifestation of increased dysregulation of oxidative stress balance or was due to increased permeability leading to increased sensitivity to stress, we performed dye uptake assays. Ethidium Bromide (EtBr) and Nile Red (NR) were used and their uptake was measured by recording the increase in fluorescence over time. The permeability studies with EtBr and NR showed increased fluorescence in KD compared to WT. The difference in fluorescence for WT and KD increased with the progress of time (Fig. 6a).

### Effect of antimycobacterial agents

The increased influx in knockdown strain compared to WT suggested a generalized mechanism, which led to increased sensitivity to pH, peroxide, and nitric oxide stresses; however, to confirm if this indeed was the case, we performed an antimycobacterial susceptibility assay and studied the inhibition of WT and KD. The findings suggested that KD compared to WT was more susceptible to inhibition by levofloxacin (LVF), streptomycin (STR) and rifampicin (RIF), but no increase in inhibition was observed against isoniazid (INH) (Fig. 6b). In addition, we studied the IlvA expression by RT-PCR after exposing *Mtb* to antimycobacterial agents. An up-regulation in IlvA expression was observed compared to untreated cells when exposed to STR, RIF, and LVF (Supplementary Fig. S5). No substantial increase in IlvA expression was noticeable upon exposure to INH. This enhanced sensitivity to STR, RIF and LVF and no increase in sensitivity to INH, as well as up-regulation of IlvA expression when exposed to STR, RIF and LVF and no substantial increase when exposed to INH, indicated mechanisms other than generalized increase in permeability being the reason of enhanced efficacy of antimycobacterial agents. All these antimycobacterial agents, which showed activity, also disturb the cellular oxidative balance^25^ and down-regulation of IlvA was somehow leading to an increased dysregulation of cellular redox balance. The increased IlvA expression when exposed to STR, RIF, and LVF further supports this assumption.

**Figure 7:**
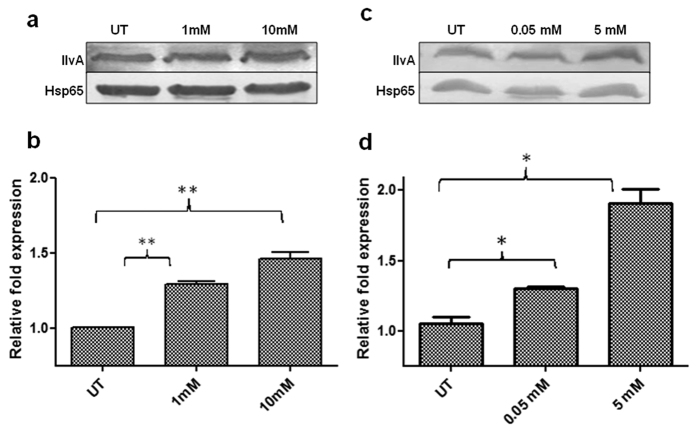
Expression of IlvA under nitric oxide and peroxide stress. Both WT and KD were exposed to different concentrations of hydrogen peroxide and DETA-NO and protein abundance was monitored by immunoblotting. (**a**,**b**) Immunoblot and densitometry of IlvA from untreated and treated with 1 mM and 10 mM hydrogen peroxide concentrations. (**c**,**d**) Immunoblot and densitometry of IlvA from untreated and treated with 0.05 mM and 5 mM DETA-NO concentrations. All densitometry data represent normalized ratio with Hsp65 used as a loading control. Results are mean ± SEM from three independent experiments performed in triplicate, significance analysis was done by Student’s *t*-test, *p < 0.05, **<0.01, ***p < 0.001.

To confirm whether IlvA expression changes in response to other stress conditions, we performed expression studies under peroxide and nitric oxide stress and results were encouraging. In the presence of hydrogen peroxide and DETA-NO a substantial increase in expression of IlvA at 1 and 10 mM hydrogen peroxide (Fig. 7a,b) was observed. In addition, considerably increased expression of IlvA was observed at 5 mM DETA-NO (Fig. 7c,d).

### Lipid profile by TLC and Mass

**Figure 8:**
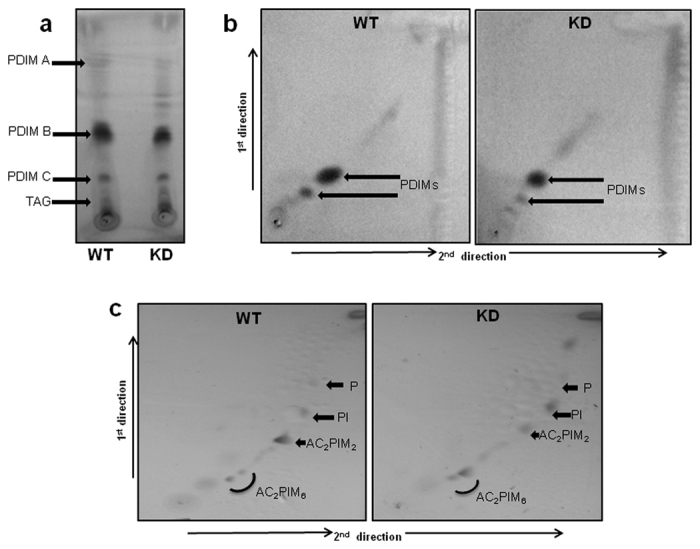
TLC analysis of lipids. (**a**) 1D TLC analysis of apolar lipids separated by petroleum ether: diethyl ether (90: 10 v/v) (**b**) 2D TLC analysis of apolar lipids from WT and KD. Lipids were separated using hexane: ethyl acetate (98:2) for 1^st^ dimension and hexane: acetone (98:2) for 2^nd^ dimension. Bands were developed by dipping in 0.2% phosphomolybdic acid dissolved in 100% ethanol and then charring at 100 °C **(c)**. The total polar lipid fraction was separated using chloroform: methanol: water (60:30:6) for 1^st^ dimension and chloroform: acetic acid: methanol: water (40:25:3:6) for 2^nd^ dimension. bands were developed using 2% orcinol in 10% H_2_SO_4_. The abbreviations PDIM, TAG, AC_2_PIM_6,_ AC_2_PIM_2,_ PI and P refer to PDIM- phthiocerol dimycocerosate, triacylglycerides, triacylglycerol, PIM- phosphatidylinositol mannosides (integers denote number of mannoside or acyl groups), PI- phosphatidylinositol and P-phospholipids respectively.

To study the effect of down-regulation on cell wall structural changes which led to enhanced permeability we performed lipid profiling of WT and KD using TLC and Mass fingerprinting. TLC studies showed an altered lipid profile in KD. PDIM B band observed in apolar fraction of KD was less intense compared to the WT; however, a diffused TAG band was visible in KD suggesting more TAG content. Apart from this, additional bands were also visible in upper region of TLC of KD (Fig. 8a,b). However, polar fraction of KD gave different distribution profile corresponding to Ac_2_PIM_6_, Ac_2_PIM_2_, PI and PE with band referring to Ac_2_PIM_2_ being less intense in KD (Fig. 8c). Mass studies of apolar and polar fractions from WT and KD showed differences in relative abundance of mass peaks. In Mass spectra, apolar fraction of KD showed relatively more abundant mass peak profile compared to WT (Supplementary Fig. S6a), while polar fraction of WT showed more abundant peaks compared to KD (Supplementary Fig. S6b).

## Discussion

Threonine dehydratases can be involved in functionally different activities as biosynthetic and biodegradative enzymes^26^. The biosynthetic threonine dehydratases are involved in biosynthesis of isoleucine by conversion of threonine to 2-oxobutanoate and release ammonia. While, biodegradative threonine dehydratase uses threonine as a carbon or nitrogen source and converts it into propionate, which may be subsequently routed into TCA cycle^27^. The KD was defective for growth on glycerol, threonine and serine as a carbon source. Also, supplementation of isoleucine restored the growth of KD on glycerol as a carbon source. This growth retardation on threonine and serine as a carbon source suggested interference in metabolizing these amino acids after down-regulation of IlvA. Although, earlier studies with *S. cerevisiae* suggest threonine’s function as a regulator of metabolic flow to methionine^28^, and studies in *Zygosaccharomyces rouxii* showed growth inhibition by causing a shortage of methionine in threonine medium^29^. However, growth defect in glycerol, serine and threonine as a carbon source by KD is not explainable by earlier reported growth inhibitions observed in the presence of threonine^28^,^29^ and appears to be due to a defect in isoleucine biosynthesis. The isoleucine concentration dependent growth restoration of KD supported its role in isoleucine biosynthesis and slow growth of KD in medium devoid of any isoleucine was due to its inability to meet its cellular requirement of isoleucine. No effect of isoleucine supplementation was observed on WT as WT was able to meet its isoleucine requirement by biosynthesizing it from simple inorganic nitrogen sources.

In the time dependent growth study, expression of IlvA was less on 21^st^ day as compared with expression on 7^th^ day of growth. In addition, expression was affected by carbon source with more expression being observed on threonine as a carbon source compared to other carbon sources. In the study to evaluate the effect of oxygen stress on expression, increase in expression intensity observed under hypoxic stress compared to aerobic growth suggested up-regulation of expression and increased expression was observed on all carbon sources. The reduced expression under partially aerobic conditions can be due to cells entering stationary phase of growth, had fewer requirements of metabolic precursors, hence lower expression levels. In the experiment with low oxygen saturation of medium, an increased expression of IlvA and DosR suggested that cells were sensing low oxygen saturation (hypoxic conditions) and hence DosR regulon was being activated as a cellular response to hypoxia and redox stress. However, simultaneous up-regulation of IlvA expression suggests its possible role in hypoxia and other stress conditions. The study of IlvA expression and effect of down-regulation under stress conditions showed a substantial increase in expression of IlvA under acidic conditions except at pH = 4.5 after 3 days. In combined pH + starvation stress study down-regulation was observed at pH = 5.5 and pH = 4.5 after 3 days. To further confirm the role of IlvA expression on cellular metabolism and overall effect on *Mtb* survival, a survival study of WT and KD was performed under pH and combined stress of pH+ starvation. While, no survival deficit between WT and KD was observed after 72 h on pH = 7.2, it was considerable at pH = 6.5 and pH = 5.5. Similarly, WT survival was higher than KD under pH+ starvation stress at pH = 6.5 and pH = 5.5. No difference was perceptible between WT and KD on pH = 4.5. The stronger expression at lower physiological pH of 5.5 and 6.5, while, no difference at pH = 4.5 suggests that *Mtb* is acclimatized to meet the environmental requirements of its physiologically required pH conditions but it fails to respond appropriately to drastic reduction in pH and enters into a non-replicating state. Also, similar response under combined stress of low pH and starvation conditions further confirms the physiologically dormant state being reached at pH = 4.5. Studies with *Mtb* and *M. bovis* BCG showed that while BCG stopped dividing at pH = 5.5, *Mtb* was still able to grow but stops dividing when pH was further lowered to pH = 4.5^30^. However, bacteria remain metabolically active and under combined pH and starvation stress it survives^31^, because of which we still get CFU counts after 72 h. *Mtb* persistence models have used low pH as an environmental condition to develop persistent cells^30^. Persistent cells become less prone to killing by stress including pH and starvation and up-regulation of IlvA at low pH or under low pH + starvation and under low oxygen saturation conditions suggests that it plays an important role in cellular stress response mechanism. Under NRP, cells respond to metabolic stress by activating certain stress responsive genes^32^.

Survival studies under nitric oxide and peroxide stress showed reduced survival of KD compared to WT with considerably increased killing being observed at higher nitric oxide and peroxide levels. Exposing *Mtb* to lower doses of nitrosating stress stops *Mtb*’s replication but at higher doses it tends to kill it by overwhelming its ability to respond to reactive nitrogen mediated stress^33^. In our study, also, we observed that at lower DETA-NO/peroxide levels *Mtb* cells remained dividing with consequent increase in CFU count but at higher doses of DETA-NO/peroxide, decreased survival was observed with more killing of KD than WT strain.

All these observations suggested that KD was more susceptible to stresses. In addition, an increase in permeability of dyes was observed. However, increased susceptibility to antimycobacterial agents such as STR, RIF and LVF and no increase in susceptibility to INH as well as increased expression of IlvA when exposed to STR, RIF and LVF at suboptimal concentrations suggests that apart from increased cellular permeability, increased susceptibility could be due to a dysregulation of cellular redox balancing system. Antimycobacterial agents such as LVF, RIF and STR are known to affect ROS and SOS responses^34^, causing oxidative imbalances during the course of their killing, while, INH being a pro-drug needs to be activated, and has no role in inducing cellular redox imbalance based killing. *M. smegmatis* recombinants with increased sensitivity to oxidative stress were shown to be more susceptible to RIF, STR and less responsive to INH^35^. Similarly, increase in expression of IlvA in response to peroxide and nitric oxide stress and decreased survival of KD under peroxide and nitric oxide stress does support our assumption of IlvA playing an important role in cellular oxidative balance.

Whole genome sequencing studies with *Mtb* clinical isolates have been used to identify the gene mutations and their relationship to resistance to antimycobacterial agents. A comprehensive knowledgebase with gene mutations in clinical isolate has been created^36^,^37^ and new and previously reported mutations have been identified in *Mtb* clinical isolates^38^, but so far, no mutation in IlvA gene has been reported. In addition, gene expression studies in clinical strains of *Mtb* have identified expression of IlvA^39^, however, no relationship of drug resistance and IlvA activity has been reported. This is the first study to report the role of IlvA under stress and its down-regulation leading to reduced survival fitness of *Mtb* under stress conditions. The lack of observed IlvA gene mutants among clinical isolates may be due to reduced survival fitness of *Mtb*.

In conclusion, we can say that threonine dehydratase (IlvA) mediated biosynthesis of isoleucine is an essential step in maintaining cellular pool of isoleucine. Also, *Mtb* threonine dehydratase down-regulation can be complemented by isoleucine supplementation. BCAAs biosynthetic pathway also plays an important role in meeting cellular requirements of Branch Chain Fatty Acids, CoA and pantothenate. This pathway becomes important in recycling BCAAs, prevents their build-up in cell, and generates Branch Chain Keto Acids (BCKAs). BCKAs can be diverted for the synthesis of metabolites to be used by TCA/methyl citrate cycle, thus provide crucial energy and carbon sources during a stage of growth when external fatty acids and carbohydrate availability is non-existent. Defective metabolism of BCKAs leads to *Mtb* being susceptible to multiple stresses^40^ as well as shows decreased virulence^41^. Disruption of *Mtb*’s BCAAs and BCKAs metabolism leads to attenuation of *Mtb* growth^42^. The increased susceptibility of KD to stress and increased expression of IlvA during stress does suggest its role in maintaining cellular oxidative balance. The down-regulation of IlvA leads to dysregulation of entire cycle involving biosynthesis and catabolism of isoleucine and subsequent redox balance involving Lpd. Lpd also functions as a peroxynitrite reductase/peroxidase and helps overcome the stress caused by host/*Mtb* generated oxidative intermediates^42^, hence, IlvA down-regulation makes *Mtb* defective in growth and vulnerable to killing due to redox dysregulation.

## Materials and Methods

### Bacterial strains, media and growth conditions

The *Mycobacterium tuberculosis* H37Ra (*Mtb*-Ra) was grown in Middlebrook 7H9 broth (MB7H9) (Difco) or Middlebrook 7H10 (MB7H10) agar (Difco), supplemented with 10% albumin-dextrose-catalase (ADC) or 10% Oleic acid-albumin-dextrose-catalase (OADC) enrichment, 0.1% glycerol and 0.05% Tween 80. *Escherichia coli* strains were grown in Luria-Bertani (LB) broth or agar plates at 37 °C and supplemented with or without ampicillin (100 μg/ml) or kanamycin (50 μg/ml). Growth studies with WT and KD were performed in Sauton’s media with glycerol being replaced by other carbon sources and supplemented with 0.05% Tween 80.

### Development and validation of knockdown strain

The IlvA gene was cloned in pMV361 in reverse orientation downstream to *hsp60* promoter. The recombinant construct *ilvA*+ pMV361 was electroporated into *M. tuberculosis* H37Ra at 2.5 kV, 36 μF and 150 Ω using a cell-electroporator (BTX). The electroporated cells were plated on MB7H11 + OADC plate supplemented with kanamycin. The recombinants were confirmed by PCR and subsequently by digestion of PCR amplicon with *Not*I restriction enzyme and sequencing. Down-regulation of expression was confirmed by immunoblotting and transcripts analysis.

### Antibody development

All experimental protocols related to animal studies were approved by CSIR-CDRI Animal Ethics Committee (approval number IAEC/2012/23N). All study methods were carried out in accordance with the approved guidelines of CPCSEA (Ministry of Environment, Forest and Climate Change), New Delhi, India. Purified protein (250 μg) mixed with incomplete adjuvant (1:1) was subcutaneously injected to female rabbit (New Zealand White, 1.5–2 kg). Booster dosing comprised of 150 μg of purified protein mixed with incomplete adjuvant and injected on 14^th^ and 28^th^ day. Blood was collected from marginal ear vein on 35^th^ day for immunoblotting and afterwards 3^rd^ booster and further immunizations were performed after periodic intervals. The experiment was terminated after 60 days and blood was collected. Antibodies were purified using antibody purification kit (Cell Biolabs).

### Transcript analysis

Down-regulation of IlvA was confirmed by both, immunoblotting as well as by transcript analysis. For transcript analysis logarithmically growing cultures (OD_600_ = 0.8) of WT and KD was pelleted at 8500 × g for 5 min. RNA isolation was performed following manufacturers protocol (TRIzol, Life Technologies). RNA samples were treated with DNase for removal of DNA contamination and quality of the RNA samples was assessed by denaturing gel (formaldehyde-agarose gel, 20% formaldehyde, 1% agarose). RNA yield was spectrophotometrically estimated (ThermoFisher, Multiskan Spectrum) and 200 ng total RNA was reverse transcribed using RevertAid first strand cDNA synthesis kit (Thermo Scientific). 20-μl reaction volume was set-up with 1 μM random hexamer primer. cDNA was used for real-time PCR study (Roche LightCycler 480) using SYBR Green (Takara).

### Growth and nutrient requirement studies

The MABA assay was used to measure the growth of KD and WT on different carbon. For this glycerol was substituted in Sauton’s media with threonine and serine, in one set glycerol was additionally supplemented with isoleucine (1 mM). MABA assay is based on colour change of Alamar blue or Resazurin. The indicator colour change from blue (oxidized state) to pink (reduced state) is used for estimation of growth and viability of cells^43^. This assay is also useful for measuring effect of toxicants on cell growth and viability. Log phase WT and KD cells (OD_600_ = 0.8) were pelleted and washed twice with phosphate buffer. The cell pellets were re-suspended in respective media and diluted to a final OD_600_ of 0.05. Media containing different carbon source and cells were distributed in 96 wells plates and incubated for 5 days at 37 °C. After completion of incubation, 25 μl of sterile resazurin (0.02%) was added to microtitre plate and absorbance was measured at 573 nm after 0, 2 and 5 h.

### Expression studies

The IlvA expression studies were performed by immunoblotting using polyclonal antibodies developed in rabbit. Expression studies were performed on different carbon sources, pH, starvation, hypoxia and under peroxide/nitric oxide stress. For pH study, WT cells grown in Sauton’s medium (with glycerol as a carbon source) till OD_600_ ~ 0.6, were harvested and washed twice with PBS (pH = 7.2). The cultures were further re-suspended in 15 ml of Sauton’s medium with glycerol as a carbon source, and medium pH adjusted at 4.5, 5.5 and 7.2, cells were incubated for 72 h (3 days) and 144 h (6 days) at 120 rpm. At the end of experiment, cells were harvested by centrifuging at 8500 rpm and used for immunoblotting. For nutrient starvation study mid exponential phase culture of WT was harvested, washed twice and re-suspended in 15 ml PBS (pH = 7.2) for 72 h and 144 h at 37 °C^44^.

The hypoxia study was performed using Wayne model^23^. For this, mid exponential phase WT cells were washed with PBS (pH = 7.2) and re-suspended in 30 ml glass tube with screw-cap and 1.0 head space ratio (HSR). Methylene blue (1.5 μg/ml) was used as an indicator dye for hypoxia and loss of colour was treated as hypoxic condition. The sample was harvested after 48 h and after colour loss was observed. These samples were treated as aerobic and hypoxic cultures. Hypoxia studies were performed with three different carbon sources and glycerol (3%) was substituted with serine (1%) and threonine (1%).

The IlvA expression was also studied under oxidative stress conditions. WT cells were used for the study. Oxidative stress was generated by adding hydrogen peroxide and DETA-NO to the culture. The concentrations used were 1 mM and 10 mM for hydrogen peroxide (Merck) and 0.05 mM and 5 mM for DETA-NO (diethylenetriamine/nitric oxide adduct; Sigma). The cells were incubated for 72 h at 37 °C. Untreated cells were used as control. Mid exponential phase cells (OD_600_ = 0.6) were taken for the study after washing them twice with PBS (pH = 7.2), followed by re-suspension in Sauton’s medium with glycerol.

The blotting studies were performed by taking cell pellet and washing it twice with 10 mM phosphate buffer (pH = 8.0) containing a protease inhibitor cocktail (10X) and re-suspended the cells in 1 ml phosphate buffer. The cells were disrupted by bead beating using 0.1 mm glass beads in Tissue Lyser II (Qiagen), supernatant was collected after centrifugation of total cell lysate at 9000 × g and 10 μg of supernatant fraction was used for electrophoresis in SDS-PAGE (12%) gel at 120 V for 90 min. The overnight transfer to PVDF membrane was performed at 25 V. For blocking 5% skimmed milk and 0.1% (v/v) tween-20 in TBS (TBST) were used. Afterwards, The PVDF membrane was incubated with anti-IlvA antibody (1:3000 dilution) in TBST buffer with 1% skimmed milk, for 6–8 hours, with shaking. Blot was washed for 15 min in TBST buffer and incubated with secondary antibody for approximately 2 h at room temperature. The detection was performed by incubating blot with DAB.

### Effect of pH and nutrient starvation on survival

The survival studies of WT and KD at acidic pH were performed in Sauton’s glycerol medium adjusted to pH = 4.5, 5.5 and 6.5. The survival studies to study the role of combined stress caused by low pH and nutrient starvation were performed in phosphate-citrate buffer adjusted to pH = 4.5, 5.5 and 6.5. The study at pH = 7.2 was treated as a positive control. Tween 80 was substituted with tyloxapol (0.05%). The cells harvested after 0 h and 72 h were plated on MB7H11 + OADC plates for CFU count^45^.

### EtBr and Nile Red accumulation/uptake studies

The cell wall permeability of WT and KD was determined by accumulation/uptake studies of EtBr and Nile Red. The experiment was performed using the protocol described by Viveiros *et al*.^46^. The brief details are as provided: log phase culture (0.8 OD) of H37Ra and recombinant strain was pelleted at 8500 rpm for 5 minutes and subsequently washed twice with PBS. The final OD_600_ = 0.4 was maintained in PBS for uptake study. The EtBr and Nile Red were used at 6 μM, 3 μM and 4 μM, 2 μM final concentration, respectively. Fluorescence was measured at black transparent 96 well fluorometric plates (Greiner Bio-One) in BioTek microplate reader. Fluorescence was recorded at 540 nm excitation and 590 nm emission at 6 minute intervals.

### Susceptibility to antimycobacterial agents

For susceptibility to antimycobacterial agents both WT and KD cells of OD_600_ = 0.05 were incubated with different concentration of antimycobacterial agents. The studies were performed in 96 well plates and plates were incubated for 5 days at 37 °C. After 5 days of incubation, 25 μl of resazurin (0.02% w/v) was added and cells were incubated for additional 5 h. Afterwards, fluorescence intensity was recorded at 544-nm excitation and 590-nm emission wavelengths (Microplate reader, BioTek Instruments). The fluorescence signal was corrected with positive control (culture alone) and negative controls (medium alone).

### Effect of oxidative stress on survival

Survival studies of WT and KD at oxidative stress conditions were performed by CFU count method after subjecting cells to different concentrations of H_2_O_2_ (Hydrogen peroxide; Merck) and DETA-NO in Sauton’s glycerol medium with 0.05% Tween 80. The H_2_O_2_ and DETA-NO concentrations used in the study were 1.0, 10 and 50 mM for H_2_O_2_; and 0.05, 1 and 5 mM for DETA-NO, respectively. Untreated cultures were taken as control for both the experiments. Sampling for both the experiments was performed after 0, 6, 24 and 72 h and cells were plated on MB7H11+OADC plates. CFU count performed after colonies were observed on plates, was used for survival calculation^47^.

### Lipid extraction and TLC analysis

For Lipid profiling of WT and KD, Log phase cultures were grown in 100 ml of 7H9+ ADC supplement media and harvested at 8000 rpm. For extraction of polar and apolar lipids, mycobacterial cells were suspended in 1:1 ratio of petroleum ether and methanolic saline (10:1) and the mixture was stirred for 2–4 h. Afterward cells were pelleted at 7000 × g and non-aqueous phase containing apolar lipids was collected in tube by clean glass pipette. The cell pellet was used for polar lipids extraction that was followed by addition of chloroform: methanol: 0.3% aqueous sodium chloride in 9:10:3 ratios. The mixture was stirred vigorously for 4–5 h and cellular debris was pelleted at 7000 × g for 10 minutes and supernatant was removed to another fresh tube. The pellet material was resuspended in chloroform: methanol: 0.3% aqueous sodium chloride in 5:10:4 ratios for 1 h. Mixture was pelleted at 7000 × g and supernatant was further collected with previous isolated supernatant. In following step chloroform and 0.3% aqueous sodium chloride was added to combined supernatant and lower layer was collected as polar lipids. Both the apolar and polar extracts were dried in pre-weighed tube using rotary vacuum evaporator^48^.

TLC analysis of apolar and polar fractions were performed following earlier prescribed methods^49^. Apolar lipid extract was separated by 1D TLC in 9:1 ratio of petroleum ether:diethyl ether and detected with 10% phosphomolybdic acid in ethanol. Polar lipid extract was separated by 2D TLC in the presence of 60:30:6 ratio of chloroform: methanol: water for 1^st^ direction and 40:25:3:6 ratio of chloroform: acetic acid: methanol: water for 2^nd^ direction. Separated polar lipids were detected with 2% orcinol in 10% sulphuric acid followed by charring.

### Mass Spectrometry

Besides TLC analysis of lipids, we also did mass studies with polar and apolar lipid fraction of WT and KD. Apolar and polar lipids fractions were dissolved in petroleum ether and 1:1 chloroform-methanol. Mass spectroscopy studies were performed by Division of Sophisticated Analytical Instruments Facility (SAIF), CSIR-CDRI and ESI-MS spectral data was recorded by using Finnigan LCQ Advantage MAX ion trap mass spectrometer (ThermoElectran Corporation). All detections were carried out in positive and negative mode.

## Additional Information

**How to cite this article**: Sharma, R. *et al*. MRA_1571 is required for isoleucine biosynthesis and improves *Mycobacterium tuberculosis* H37Ra survival under stress. *Sci. Rep*. **6**, 27997; doi: 10.1038/srep27997 (2016).

## Supplementary Material

Supplementary Information
