# Supplementary material for: MRA_1571 is required for isoleucine biosynthesis and improves Mycobacterium tuberculosis H37Ra survival under stress
Source: Sci Rep. 2016 Jun 29;6:27997. doi: 10.1038/srep27997 (PMC4926081; doi:10.1038/srep27997)

# MRA_1571 is required for isoleucine biosynthesis and improves *Mycobacterium tuberculosis* H37Ra survival under stress

**Rishabh Sharma[1], Deepa Keshari[1], Kumar Sachin Singh[1], Shailendra Yadav[1] and Sudheer Kumar Singh[1, 2#]**

**Supplementary Figures**

**Supplementary Figure S1. Confirmation of antisense construct.** (**a**) Shows confirmation of *ilvA* antisense construct in pMV361 by digestion with *Xho*I. (**b**) PCR amplification of KD genomic DNA with *ilvA* reverse (downstream of gene) primer and reverse *aph* (downstream Kanamycin) primer. (**c**) Restriction digestion of PCR amplicon by *Not* I. (**d**) Schematic representation of KD construct in pMV361.

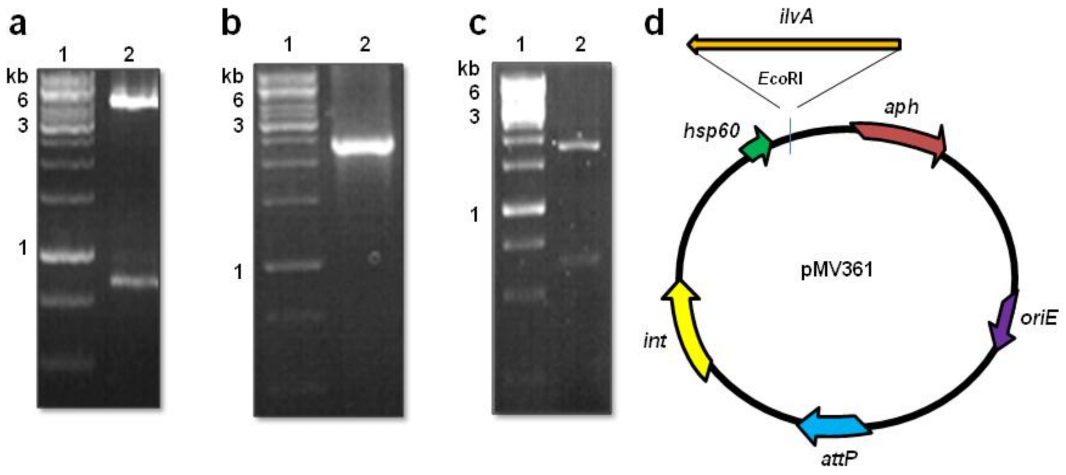

**Supplementary Figure S2. Antibody development and knockdown confirmation.** (**a**) Immunoblotting of threonine dehydratase with raised antibody in rabbit. Lane 1 is immunoblot with 0-day rabbit serum, Lane 2 is immunoblot with 35-day rabbit serum. (**b-c**) Confirmation of down-regulation by immunoblotting and densitometry analysis. (**d**) Confirmation of down-regulation by transcript analysis. Results are mean ± SE from three independent experiments performed in triplicate, significance analysis was done by Student's *t*-test, **<0.01, ***$p$<0.001.

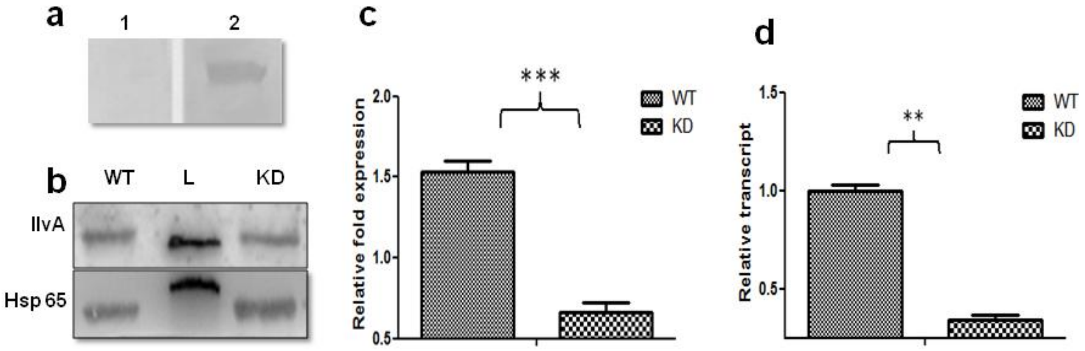

**Supplementary Figure S3. Effect of isoleucine concentration on growth.** Growth studies were performed by MABA using different concentrations of isoleucine for growth of both wild-type (WT) and knockdown (KD). The resazurin fluorescence measurements were recorded at 530 (excitation) / 590 (emission) and change in resazurin fluorescence was used as an indicator of growth. The fluorescence changes from untreated WT sample were used for normalizing the growth in isoleucine treated WT, KD and untreated KD samples. The growth study was performed in Sauton's medium with glycerol as a carbon source and ammonium chloride as a nitrogen source. Isoleucine was additionally supplemented in concentrations ranging from 0.25mM to 4mM. Results are mean ± SE of three independent experiments performed in triplicate.

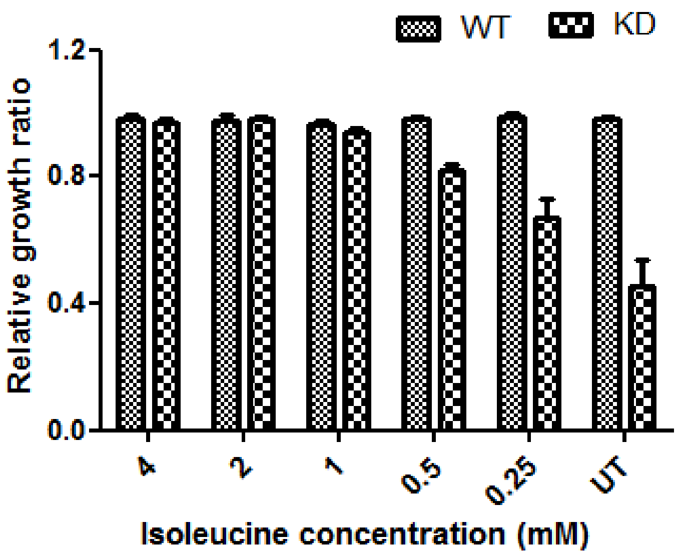

**Supplementary Figure S4. Effect of oxygen saturation on expression of IlvA**. Expression of IlvA was studied at 48h and under hypoxic conditions by quantitative real-time PCR (RT-PCR). Methylene blue colour loss was used as an indicator for hypoxia and IlvA as well as DosR transcript levels were studied. 16s rRNA was used as a reference. IlvA and DosR transcript levels under hypoxic conditions were normalized with transcript levels at 48h. WT refers to wild-type *Mtb*. Bar graph represents IlvA and DosR transcript levels in WT at 48h and under hypoxic conditions. Results are mean ± SE of three experiments performed in triplicate.

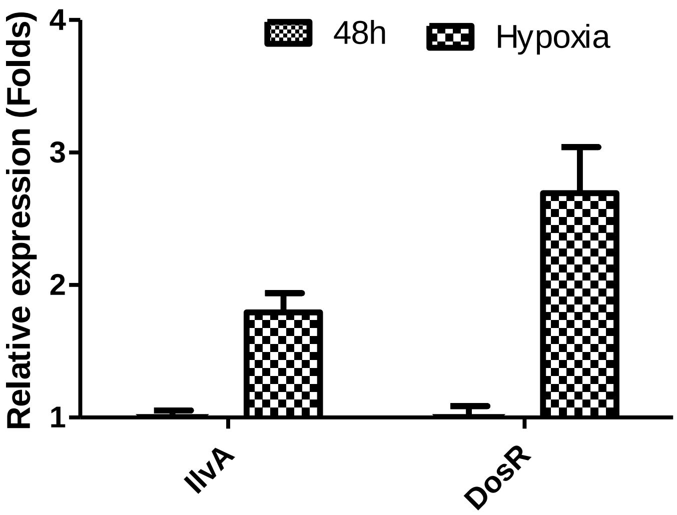

**Supplementary Figure S5. Effect of antimycobacterial agents on expression of IlvA.** Quantitative real time PCR (RT-PCR) based expression profiling of IlvA was performed at 10 fold (10 F) and 20 fold (20 F) dilution from MIC of respective antimycobacterial agents. The experiments were performed in MB7H9 + ADC medium and sampling was performed after 48h. INH, STR, RIF and LVF refer to isoniazid, streptomycin, rifampicin and levofloxacin. 16S rRNA was used as a reference and relative expression folds were normalized with expression level from untreated sample. Results are mean ± SE of three independent experiments performed in triplicate.

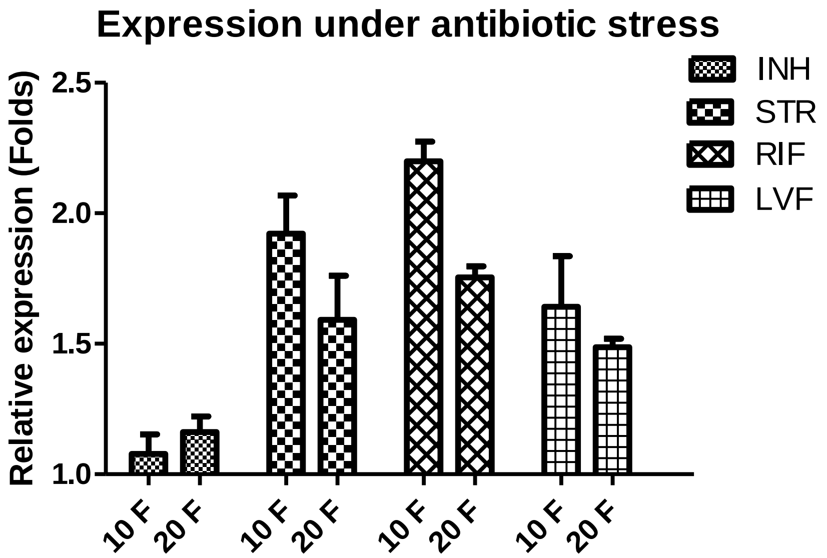

**Supplementary Figure S6. Mass profiling of lipids.** (**a**) ESI-MS spectra recorded for apolar lipid extracts from wild-type (WT) and knockdown (KD) (**b**) ESI-MS spectra recorded for polar lipid extracts from WT and KD. Spectra are representative of a set of experiments performed in three independent sets.

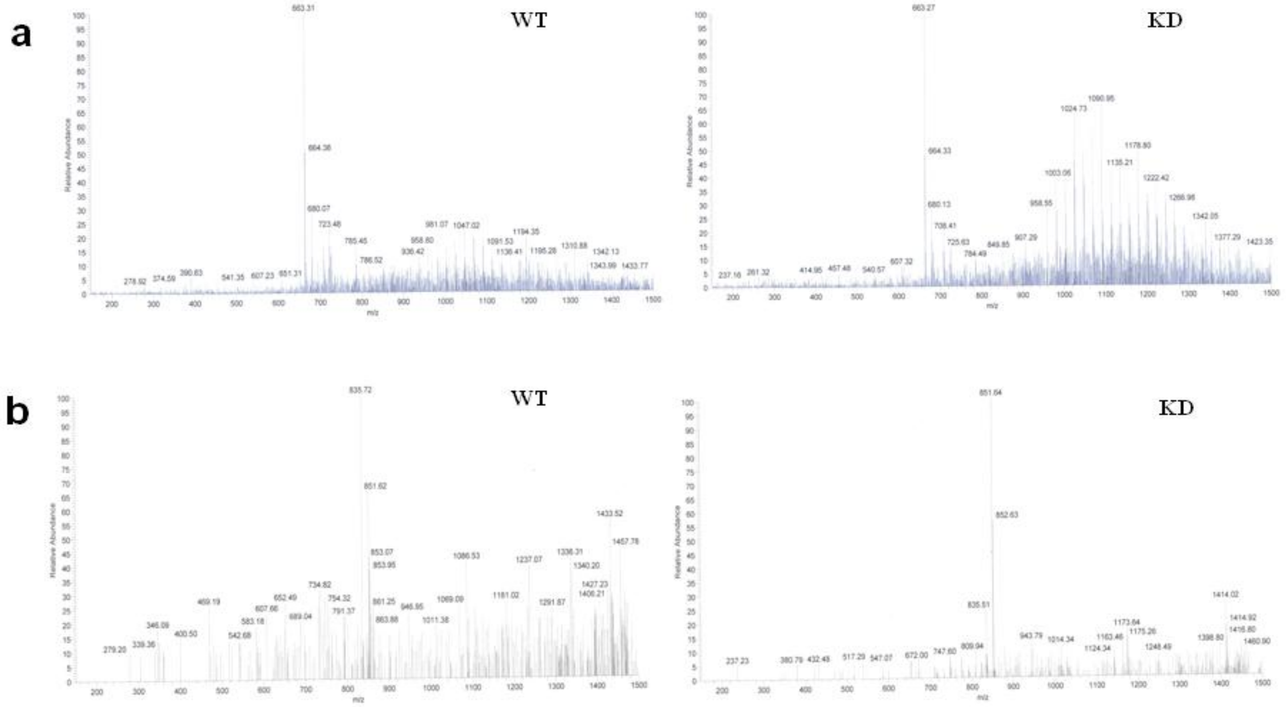

Supplement: Supplementary Information [file srep27997-s1.pdf]
